# Dupilumab-Induced Psoriasis in a Patient With Prurigo Nodularis: A Case Report

**DOI:** 10.7759/cureus.81636

**Published:** 2025-04-03

**Authors:** Hiroki Ogawa, Kentaro Izumi

**Affiliations:** 1 Dermatology, KKR Sapporo Medical Center, Sapporo, JPN

**Keywords:** a case report, drug induced psoriasis, dupilumab, prurigo nodularis (pn), psoriasiform dermatitis, rare side effect

## Abstract

Dupilumab is a monoclonal antibody targeting the interleukin-4 (IL-4) and interleukin-13 (IL-13) receptor complex and is used for the treatment of atopic dermatitis (AD), severe asthma, chronic rhinosinusitis with nasal polyps, and prurigo nodularis (PN). Dupilumab has shown high efficacy in PN; however, adverse effects such as conjunctivitis, injection site reactions, and pharyngitis have been reported. Meanwhile, dupilumab-induced psoriasis is a relatively rare adverse effect. Here, we present a case of a 71-year-old woman with a seven-year history of PN that was refractory to topical corticosteroids and narrow-band ultraviolet B (UVB) therapy. The patient received dupilumab injections every two weeks, resulting in an improvement in pruritic nodules. However, three months after the initiation of dupilumab, multiple scaly erythematous plaques appeared on the trunk and extremities. Skin biopsy revealed parakeratosis, regular acanthosis with epidermal thickening, loss of the granular layer, and subcorneal neutrophilic infiltration. Based on these findings, the patient was diagnosed with dupilumab-induced psoriasis. After discontinuing dupilumab and initiating topical corticosteroid therapy, the psoriatic lesions showed significant improvement within three months. Psoriasis induced by dupilumab has been documented in multiple reports involving patients with atopic dermatitis. Only one case of dupilumab-induced psoriasis in a PN patient has been reported, which was limited to the scalp. To the best of our knowledge, this case represents the first report of extensive psoriasiform lesions on the trunk and extremities in a PN patient. While mild cases may be managed without discontinuing dupilumab, severe cases may require cessation of the drug. This case highlights the first report of extensive psoriasiform lesions in a PN patient with dupilumab. Dermatologists should be aware that dupilumab can induce psoriasiform lesions in PN patients and should conduct careful monitoring during treatment. Additionally, discontinuation of dupilumab should be considered in severe cases.

## Introduction

Dupilumab is an injectable biologic agent effective for the treatment of severe atopic dermatitis (AD), severe bronchial asthma, chronic rhinosinusitis with nasal polyps, and prurigo nodularis (PN). This drug is a human monoclonal antibody of the immunoglobulin G4 (IgG4) subclass that specifically binds to the interleukin-4 (IL-4) and interleukin-13 (IL-13) receptor complex, thereby inhibiting their signaling pathways [1]. The de novo development of psoriasis is an adverse effect of dupilumab [2].

Previous literature has reported that psoriasis can occur in AD patients treated with dupilumab. However, reports of dupilumab-induced psoriasis in PN patients are rare, with only one documented case of localized psoriatic lesions to the best of our knowledge. Here we highlight the potential for widespread psoriasis as a rare but significant adverse event even in PN patients treated with dupilumab.

## Case presentation

**Figure 1:**
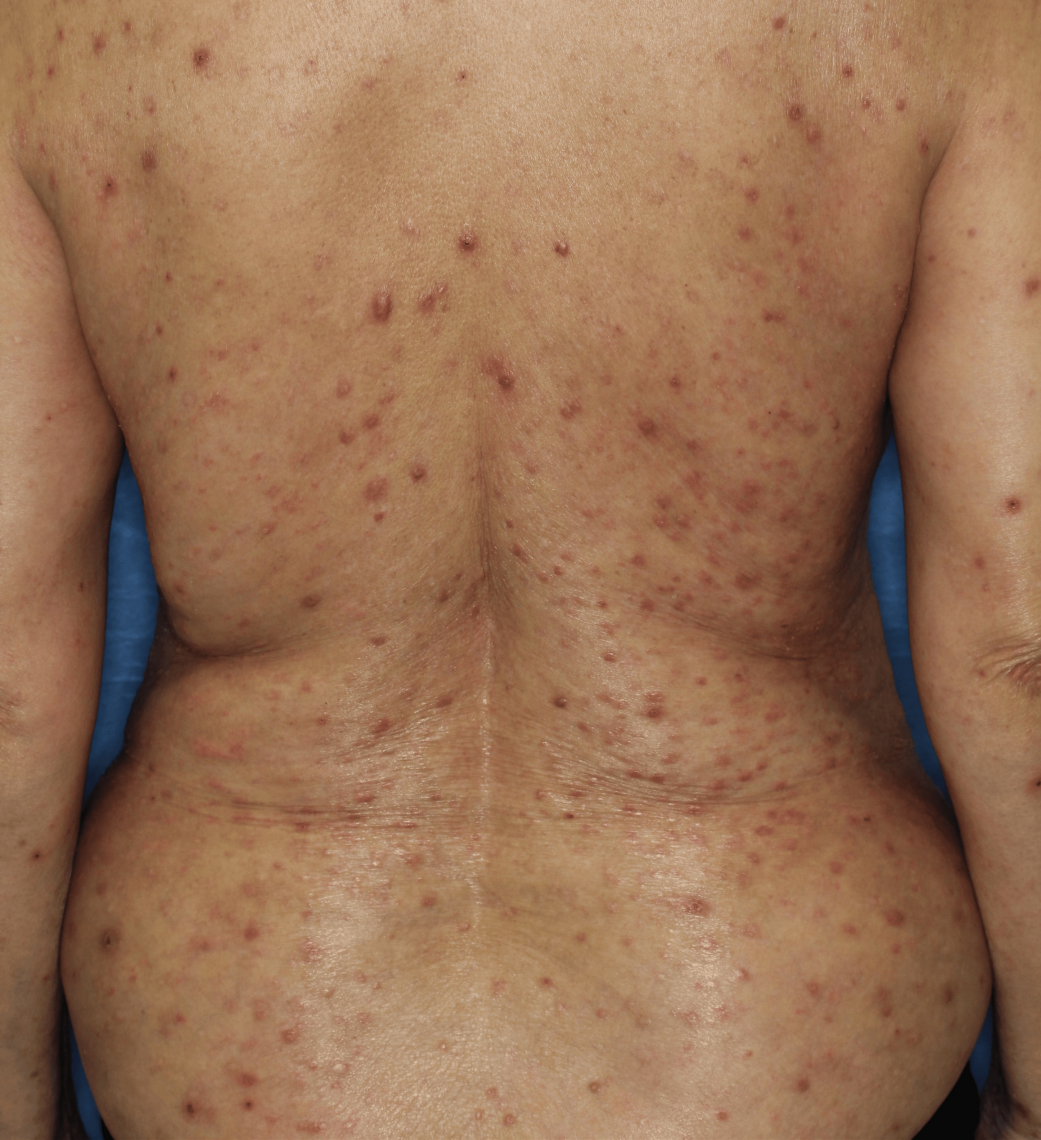
Physical findings at initial presentation Multiple firm reddish nodules on the trunk at the initial evaluation.

**Figure 2:**
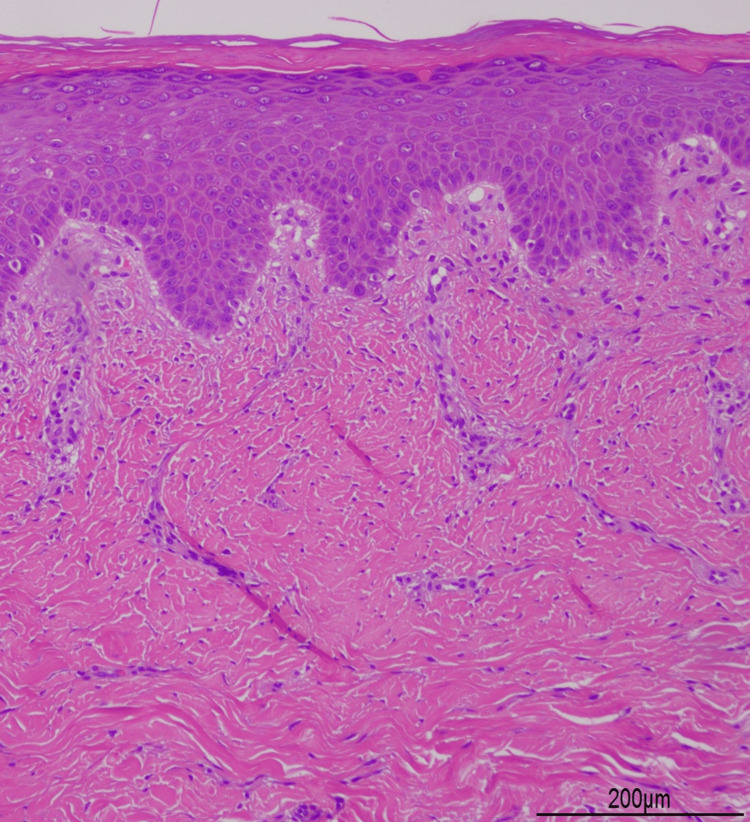
Histopathological examination of the nodule Histopathological examination of the nodule on the back revealed compact orthokeratosis, hypergranulosis, acanthosis, and superficial perivascular lymphocytic infiltration (100× magnification).

A 71-year-old female presented with a seven-year history of itchy reddish nodules (Figure 1). She did not have any past medical history. She had been treated at a nearby clinic with topical steroids and antihistamines, but her lesions showed little improvement. Physical examination showed multiple firm nodules on her trunk and extremities. A skin biopsy specimen from a nodule on her back revealed compact orthokeratosis, hypergranulosis, acanthosis, and superficial perivascular lymphocytic infiltration (Figure 2). The patient was diagnosed with PN.

**Figure 3:**
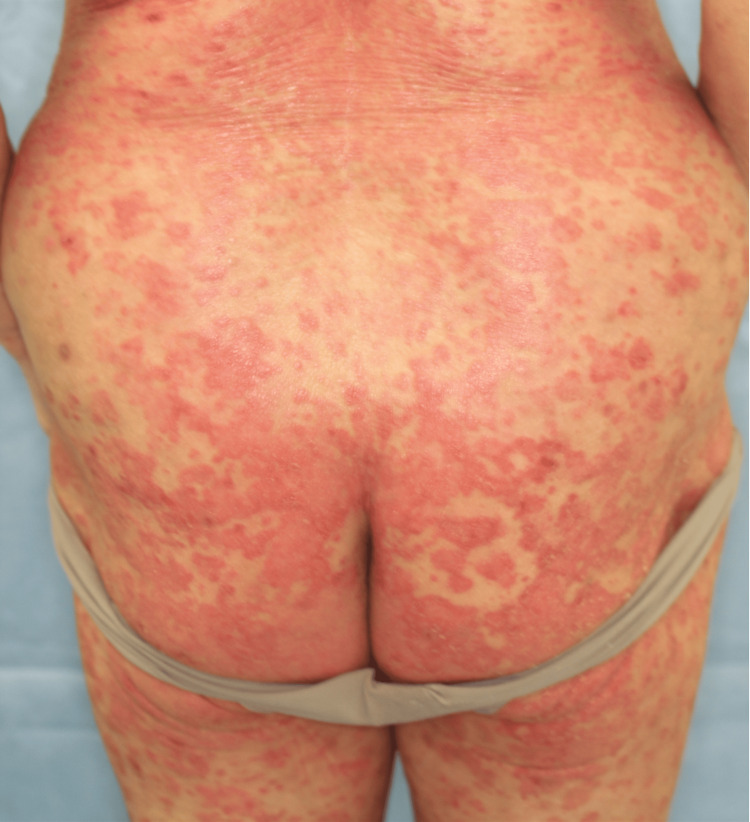
Physical findings three months after initiation of dupilumab therapy Patient’s trunk after three-month treatment with dupilumab. There were multiple scaly erythema on her trunk and extremities.

As skin lesions were refractory to 0.05% clobetasol propionate ointment and narrow-band ultraviolet-B phototherapy for 12 months, dupilumab was administered every two weeks. After eight doses of dupilumab, pruriginous nodules improved, whereas multiple scaly erythematous lesions developed on her trunk and extremities three months after dupilumab administration (Figure 3).

**Figure 4:**
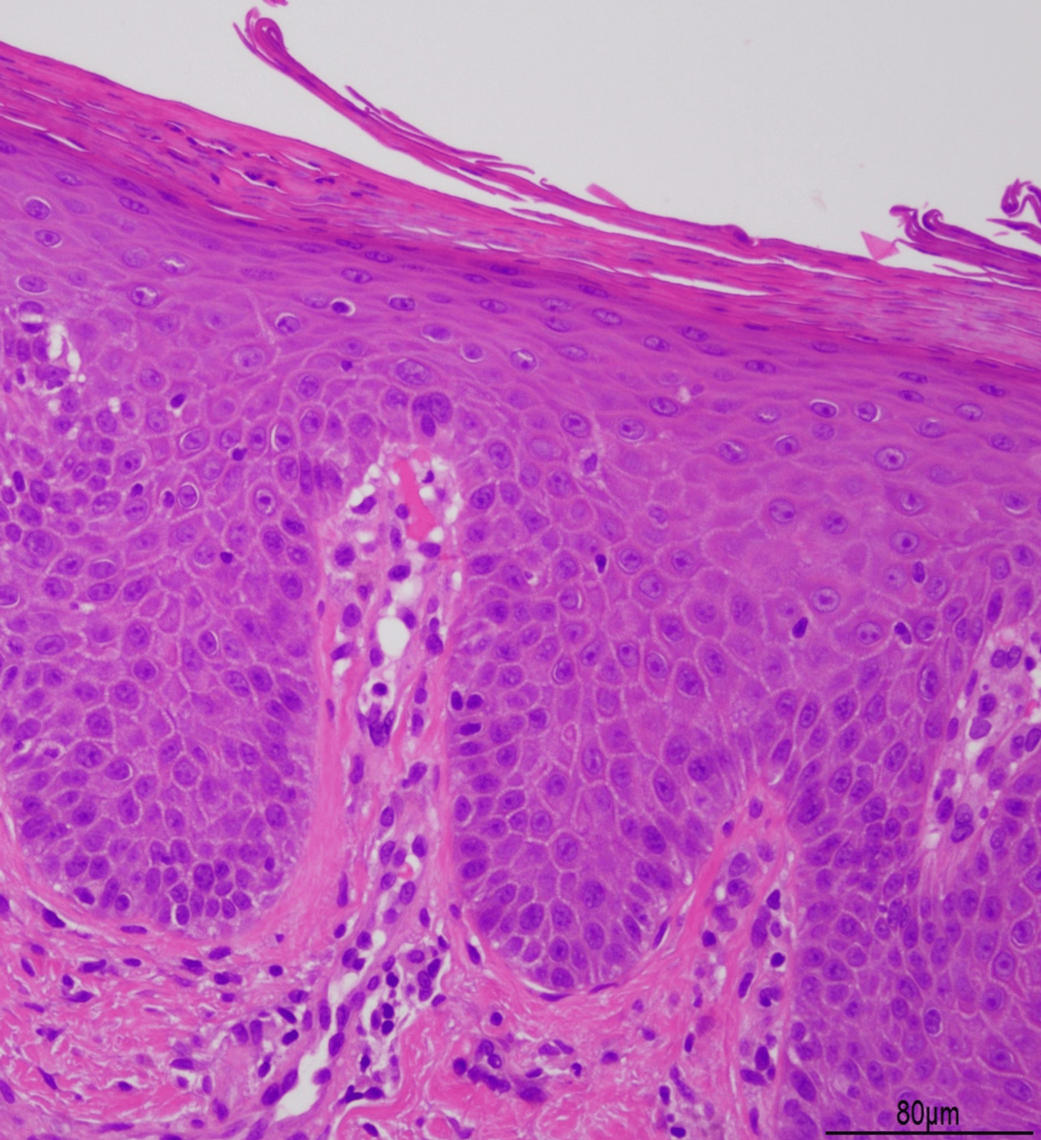
Histopathological findings of the erythema Histopathological examination of the scaly erythema on the trunk showed parakeratosis, regular acanthosis, and lack of granular layer. Neutrophil infiltration was observed in the stratum corneum (100× magnification).

Histopathological examination of scaly erythema on her trunk revealed parakeratosis, regular acanthosis, a lack of granular layer, and neutrophil infiltration below the stratum corneum (Figure 4).

**Figure 5:**
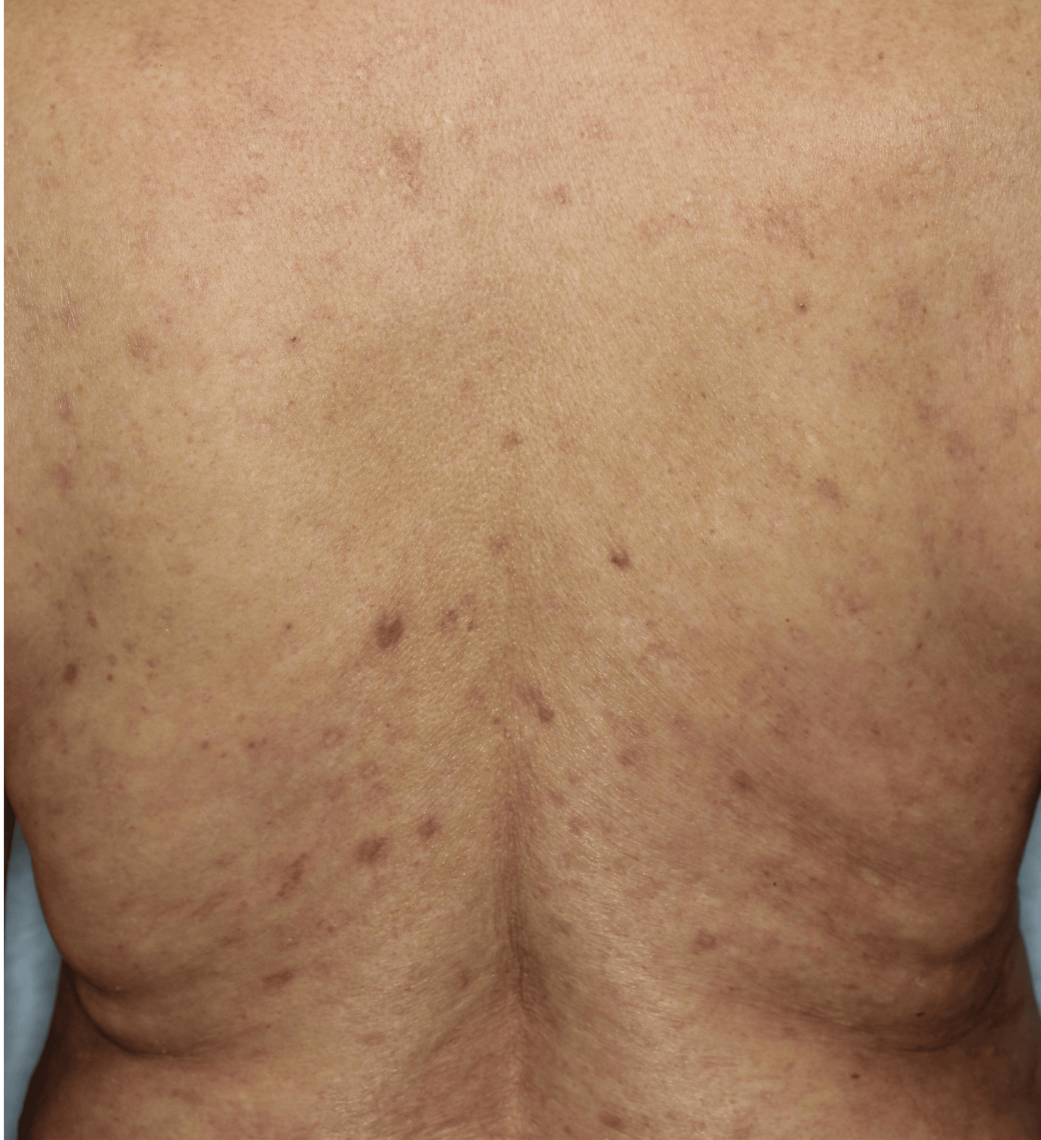
Physical findings after treatment Significant resolution of scaly erythematous lesions on the back after three months of discontinuing dupilumab and using clobetasol propionate 0.05% ointment.

Based on the above clinical and histopathological findings, the patient was diagnosed with dupilumab-induced psoriasis. We then ceased dupilumab and treated her with 0.05% clobetasol propionate ointment. Psoriatic plaques showed remarkable improvement in three months (Figure 5).

## Discussion

PN is a chronic skin disorder clinically characterized by pruritic, firm nodules preferentially distributed on the extensor surfaces of the extremities. Potent topical corticosteroids, intralesional steroid injection, and antihistamines are used for first-line therapy for PN to interrupt the itch-scratch cycle. Ultraviolet (UV) light therapy and/or systemic immunosuppressants are adopted as a second-line therapy [3]. However, these treatment modalities have limited efficacy, resulting in inadequate disease control. In 2022, the FDA approved dupilumab as a systemic treatment indicated for adult PN. Because a double-blind, randomized, placebo-controlled phase 3 clinical trial demonstrated significant improvement in both itch and skin lesions in PN patients treated with dupilumab [4], the drug is expected to improve the prognosis for refractory cases of PN. Regarding adverse effects of dupilumab, conjunctivitis, injection-site reactions, nasopharyngitis, and headaches are reported as the side effects associated with dupilumab [5]. However, dupilumab-induced psoriasis remains relatively rare. The incidence rates of dupilumab-induced psoriasis range from 1.8% to 3.3% in AD patients treated with dupilumab [2].

Regarding the underlying mechanisms, the onset of dupilumab-induced psoriasis could be explained by Th polarization differences between PN and psoriasis. Psoriasis primarily features Th1/Th17 cells, whereas PN involves increased levels of the Th2 cytokines IL-4 and IL-13 [6]. Dupilumab inhibits Th2-mediated inflammation, which may have led to an immunologic shift toward the Th1/Th17 pathway [2,7]. To date, there is only a PN case developing dupilumab-induced psoriasis reported by Al Hawsawi et al. In this case, the psoriatic skin lesion was limited to the scalp [8]. In contrast, our case is the first report, to the best of our knowledge, of a PN patient developing dupilumab-induced psoriasis with widespread psoriatic skin lesions on the trunk and extremities. While dupilumab discontinuation may not be essential for managing mild to moderate dupilumab-induced psoriasis, topical glucocorticoids alone or combined with vitamin D derivatives are commonly utilized, similar to the treatment approaches for classic mild psoriasis. In cases of severe dupilumab-induced psoriasis, however, cessation of the drug is often considered.

## Conclusions

In a PN patient, we report the first case, to the best of our knowledge, of widespread dupilumab-induced psoriasis affecting the trunk and extremities. PN is a skin disorder characterized by multiple nodules with intense pruritus, often difficult to control with conventional treatments. Dupilumab is an effective treatment option for PN. As its use increases, not only common adverse effects like upper respiratory tract infections and conjunctivitis, but also rare complications such as the de novo development of psoriasis seen in this case, may become more frequent. Dermatologists should be aware that dupilumab may cause psoriatic skin lesions even in PN cases, which may necessitate its discontinuation.
